# LONELINESS AND RISK OF ELDER ABUSE: DOES SIZE OF SOCIAL NETWORK MAKE A DIFFERENCE?

**DOI:** 10.1093/geroni/igae098.2064

**Published:** 2024-12-31

**Authors:** Louis To, Jasmin He, Lok Man Leung, Ronald Tsui, Debby Wan, Elsie Yan

**Affiliations:** The Hong Kong Polytechnic University, Hong Kong, Hong Kong, China (People’s Republic); The Hong Kong Polytechnic University, Hong Kong, Hong Kong, China (People’s Republic); The Hong Kong Polytechnic University, Hong Kong, Hong Kong, China (People’s Republic); Lingnan University, Hong Kong, Hong Kong, China (People’s Republic); The Hong Kong Polytechnic University, Hong Kong, Hong Kong, China (People’s Republic); The Hong Kong Polytechnic University, Hong Kong, Hong Kong, China (People’s Republic)

## Abstract

Elder abuse is a serious public health issue. Research demonstrated that loneliness is a risk factor for abuse. The present study examined the association between loneliness and elder abuse in a convenient sample of 686 community dwelling older persons in Hong Kong. Participants were recruited from senior community centers across the territory. They provided information on their demographic characteristics, sense of loneliness (DJGLS-6), social network (LSNS-6), and risk of elder abuse (EASI and VASS-10). 37.4% of the participants were screened positive on EASI or VASS-10. Results of logistical regression show that participants who were female (OR=-.505), married (OR=-.951), of a younger age (OR=-.044), reported greater sense of loneliness (OR=.266) were more likely to report risk of being abused. Co-residence with a family member and size of social network was not significant in the model (p>.05). Multiple regression analysis was conducted to identify factor associated with sense of loneliness. Participants of a younger age (Beta=-.019), living alone (Beta=-.170), and had a smaller social network (Beta=.-085) reported greater sense of loneliness. Findings from the present study adds to the larger literature on the impact of various demographic factors and sense of loneliness on elder abuse. Prevention and intervention efforts should focus on female young-olds in a marital relationship. Efforts to mitigate sense of loneliness could potentially help tackle elder abuse. Although increasing social network size does not have a direct impact on risk of elder abuse, it could potentially mitigate sense of loneliness thereby decrease risk of abuse.

